# Role of Maternal and Child Health Handbook on Improving Maternal, Newborn, and Child Health Outcomes: A Systematic Review and Meta-Analysis

**DOI:** 10.3390/children10030435

**Published:** 2023-02-23

**Authors:** Etsuko Nishimura, Md Obaidur Rahman, Erika Ota, Noriko Toyama, Yasuhide Nakamura

**Affiliations:** 1 Graduate School of Nursing Science, St. Luke’s International University, Tokyo 104-0044, Japan; 2 Center for Surveillance, Immunization, and Epidemiologic Research, National Institute of Infectious Diseases, Tokyo 162-8640, Japan; 3 Center for Evidence-Based Medicine and Clinical Research, Dhaka 1230, Bangladesh; 4 Tokyo Foundation for Policy Research, Tokyo 106-6234, Japan; 5 Faculty of Medicine, University of the Ryukyus, Okinawa 903-0215, Japan; 6 Friends of WHO Japan, Osaka 540-0029, Japan

**Keywords:** public health, maternal and child health, systematic review

## Abstract

The objective of this review is to assess and synthesize the role of the maternal and child health (MCH) handbook on improving healthcare service utilization, behavior change, and health outcomes for women and children. A systematic search of all relevant existing reports was conducted on 14 January 2021, using the following online bibliographic databases: PubMed, EMBASE, MEDLINE, The Cochrane Library, Academic Search Premier, Emcare, APA PsycINFO, and Web of Science. Two reviewers independently performed study selection, data extraction, and quality assessment. We included 7 trials from 1430 articles, and a total of 2643 women. As overall risk of bias assessment, most domains of the Cochrane risk-of-bias assessment tool showed a high or unclear risk of bias. The risk of ≥6 antenatal care (ANC) visits was 19% higher (RR 1.19, 95% CI 1.09 to 1.30, I2 = 47%, 2 studies, 955 women, moderate certainty of evidence) and skilled birth attendants during delivery was 13% higher (RR 1.13, 95% CI 1.04 to 1.24, I2 = 0%, 2 studies, 1094 women, low certainty of the evidence) in the intervention group than in the control group. The MCH handbook can increase maternal health service utilization and early breastfeeding practice. It also leads to a sense of autonomy during ANC, better communication with healthcare providers, and support from family members.

## 1. Introduction

High maternal and neonatal mortality are global public health issues, especially in low- and middle-income countries. These deaths can be prevented by providing effective care to all newborns and women who give birth in health facilities, closing the quality gap [1]. The maternal and child health (MCH) handbook is a tool that can promote continuity of care from the beginning of pregnancy and improve the quality of healthcare. The MCH handbook is designed to keep all records of health conditions with health advice or education for a woman and her child into a single document during pregnancy, delivery, and the postpartum period, such as maternal care and the child’s growth pattern and vaccination schedule [2,3,4]. The MCH handbook is being considered as a comprehensive and consistent recording tool of maternal, newborn, and child health services for pregnant or postpartum women and for health service providers. The use of the MCH handbook helps health service providers deliver appropriate MCH services following set standards and enables them to record medical information (e.g., test results) properly and accurately. It can support improvements in the continuum of care [5,6]. As a result, the MCH handbook has been attracting more attention from health ministries and professional organizations as an effective tool for promoting a life course approach to healthcare [3].

Despite the potentially significant benefits, only a few countries provide the MCH handbook nationwide. This might be due to a lack of rigorous evaluation on assessing the effectiveness of the MCH handbook, as there is a lack of high-quality studies to show its superiority over existing alternatives [7,8]. Therefore, a rigorous evaluation of high-quality studies is needed to assess the effect of the MCH handbook on improving maternal, newborn, and child health outcomes. Several systematic reviews on the MCH handbook have been published [8,9,10,11,12,13], four of them with meta-analyses [8,9,12,13]. However, only one meta-analysis restricted to randomized controlled trials was published in 2015 [9]. Since 2015, papers from randomized controlled trials on the MCH handbook have been published, and the evidence needs to be updated. Thus, this review conducts a meta-analysis of randomized controlled trials that provided all MCH handbook types, including a card format and electronic records, as interventions. The objective of the review is to assess and synthesize the role of the MCH handbook in improving healthcare service utilization, behavior change, and health outcomes for women and children.

## 2. Materials and Methods

The systematic review protocol was registered on PROSPERO (register number: CRD42021267171) [14]. We followed the guidelines of the Cochrane Handbook for Systematic Reviews of Interventions to conduct the review [15]. The findings of the review were reported according to the preferred reporting items for systematic review and meta-analysis (PRISMA) guidelines [16] (Table S1).

### 2.1. Criteria for Considering Studies in This Review

We defined the following study eligibility criteria:

(i) Participants

We included studies conducted on pregnant women from the period between their first antenatal visits and the end of the postpartum period.

(ii) Interventions

We considered interventions providing any forms of MCH handbooks (e.g., booklets, cards, home-based records, paper-based records, electronic records, and case notes) that focused on improving maternal, newborn, and child health outcomes.

(iii) Comparators

We included those studies that assessed the effectiveness of MCH handbooks with usual care or not using MCH handbooks, excluding studies comparing different forms of MCH handbooks.

(iv) Outcomes

We included studies that reported maternal, newborn, and child health outcomes. We categorized them as primary and secondary outcomes listed in the following section.

(v) Study designs

We considered individual randomized controlled trials (RCTs) and cluster-RCTs.

### 2.2. Outcomes of Interests

The following maternal, newborn, and child health outcomes have been reported in this systematic review.

#### 2.2.1. Primary Outcomes

(i) Number of antenatal care (ANC) visits;
(ii) Number of facility deliveries;
(iii) Skilled birth attendance (SBA) at the time of delivery;
(iv) Number of postnatal care (PNC) visits for mother;
(v) Number of cesarean deliveries;
(vi) Proportion of exclusive breastfeeding practices;
(vii) Number of pregnant women who stopped or reduced cigarette smoking.

#### 2.2.2. Secondary Outcomes

(i) Maternal satisfaction and control

Number of women who felt in control and involved in decision making during their pregnancy/who reported that they were satisfied with their ANC/who wanted to carry their MCH handbook in a subsequent pregnancy.

(ii) Maternal and child vaccination coverage

Number of pregnant women who took a full dose of tetanus vaccine;

Number of children who were fully vaccinated (Polio, diphtheria-pertussis-tetanus (DPT), measles, and so on).

(iii) Partner involvement in the pregnancy, during labor, and after childbirth

Number of partners that attended ANC visit/who presented during labor/actively involved in the care of newborns.

(iv) Maternal morbidity and mortality

Number of women who were seeking care for postpartum complications/who had postnatal depression/ who had short-term morbidity (hemorrhage, infection, blood transfusion, pregnancy loss, and intensive care unit admission).

Number of maternal deaths

(v) Infant morbidity and mortality

Number of women who were seeking care for their newborn illness;

Number of neonates admitted to intensive/special care unit, neonatal deaths, including stillbirths;

Other adverse birth outcomes such as stunting, wasting, underweight, and low birth weight.

(vi) Administrative outcomes

Availability of complete antenatal records at the time of delivery;

Number of MCH handbooks lost or left at home when attending clinic/hospital.

### 2.3. Study Identification

We searched the following electronic databases from the inception to 14 January 2021, using the comprehensive search strategy described in Table S2: PubMed, EMBASE, MEDLINE, The Cochrane Library, Academic Search Premier, Emcare, APA PsycINFO, and Web of Science. The search terms involved Medical Subject Headings (MeSH), title/abstract (ti/ab), and text words (tw). We did not limit the search by language, date, or publication type.

The reference lists of relevant systematic reviews and primary studies were checked to identify additional studies that were not captured by the electronic searches.

### 2.4. Study Selection, Data Extraction and Quality Assessment

Two reviewers independently performed each step below and cross-checked information. Disagreements were resolved through discussion or a third reviewer when required.

Using the predefined study eligibility criteria, the initial screening of the titles and abstracts of the retrieved studies from all databases was performed using the Rayyan QCRI tool, an online platform for study screening, and excluded irrelevant studies [17]. Next, the reviewers selected all potentially relevant studies for full-text screening and critically assessed their eligibility in detail. They also checked reference lists of all relevant systematic reviews and included primary studies. We used a study flow diagram (PRISMA flow diagram) to describe the study selection process, including the number of studies identified, excluded, and included in this review.

We used a standardized data extraction form that contained the following information: author information, year of publication, study design, setting and country of the study, study period, number of participants, characteristics of participants, details of intervention and control, types of outcome measures, and study results. The data extraction form and other materials generated during the current review are available from the corresponding author upon reasonable request.

The risk of bias of the included study was assessed using the Cochrane risk-of-bias 1.0 assessment tool [18].

### 2.5. Data Synthesis and Analytical Approach

We summarized the key characteristics of the included studies in a table (mainly study characteristics, participant characteristics, intervention characteristics, and key findings of studies). After obtaining a sufficient number of studies, we used pair-wise meta-analysis to summarize the intervention effects for each outcome separately. We used I^2^ statistics to measure the heterogeneity of the included studies in the meta-analysis and interpret them by following the definitions in the Cochrane Handbook for Systematic Reviews of Interventions [15]. Having substantial heterogeneity among the included studies, we used random-effects meta-analysis. Otherwise, a fixed-effect meta-analysis was used to pool the effect sizes. Meta-analysis results were presented in forest plots. We used the risk ratio (RR) for dichotomous data and the mean differences (MD) for continuous data with corresponding 95% confidence intervals (CI). However, when there were insufficient studies to perform a meta-analysis of an outcome, we narratively presented the study findings for that outcome. When the necessary data were available, we performed subgroup analysis based on the study population, settings, interventions, or study designs. If a meta-analysis included at least ten studies, we assessed publication bias using funnel plots and the Egger’s test. Sensitivity analysis was performed if studies had high selection bias and attrition bias.

### 2.6. Assessment of the Certainty of the Evidence

We assessed the certainty of evidence for the effectiveness of the MCH handbook on outcomes by the grades of recommendations assessment, development, and evaluation (GRADE) approach.

### 2.7. Patient and Public Involvement

It was not appropriate or possible to involve patients or the public in the design, conduct, reporting, or dissemination plans of our research.

## 3. Results

### 3.1. Study Inclusion

**Figure 1:**
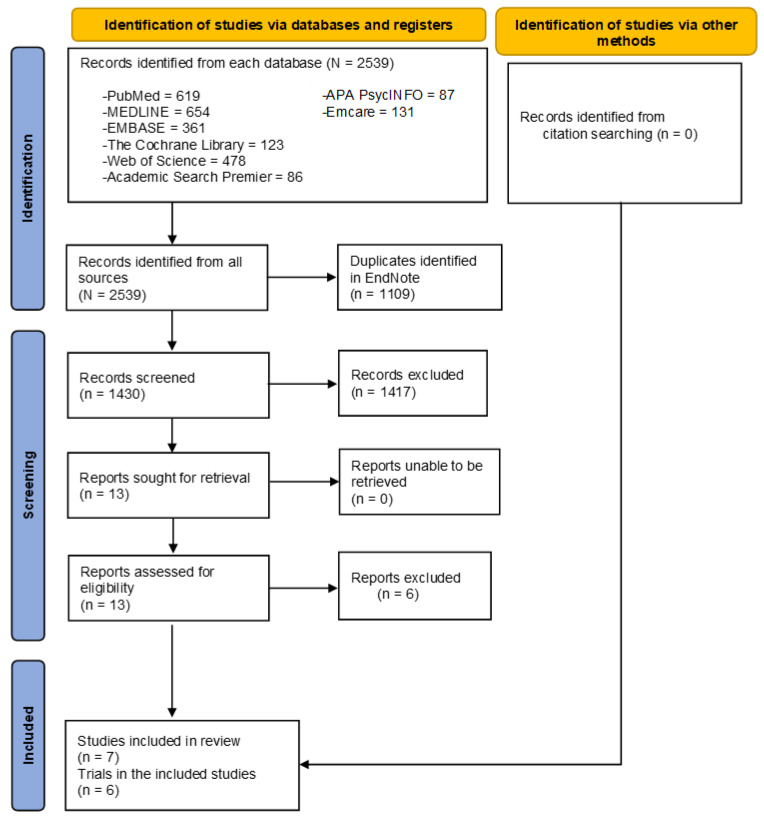
PRISMA study flowchart.

A total of 1430 non-duplicate articles identified from all targeted electronic databases and other resources underwent initial title and abstract screening. We excluded 1417 articles in the initial screening and assessed the remaining 13 articles for detailed study eligibility. In the full-text screening, five articles were excluded that did not meet the study eligibility criteria. The main reasons for exclusion were incorrect interventions and study designs. Finally, we identified seven articles from all resources suitable for this review. The study selection process was reported in the PRISMA study flow diagram (Figure 1).

### 3.2. Characteristics of the Included Studies

The studies were conducted in Australia, Indonesia, Iran, Mongolia, and the United Kingdom and published between 1987 and 2019. A total of 1981 pregnant women, including mothers with their infants, participated in the studies.

Different forms of interventions were provided to pregnant women or mothers with their infants. The detailed characteristics of intervention in the included studies are presented in Table S3.

### 3.3. Overall Risk of Bias Assessment of the Included Studies

**Figure 2:**
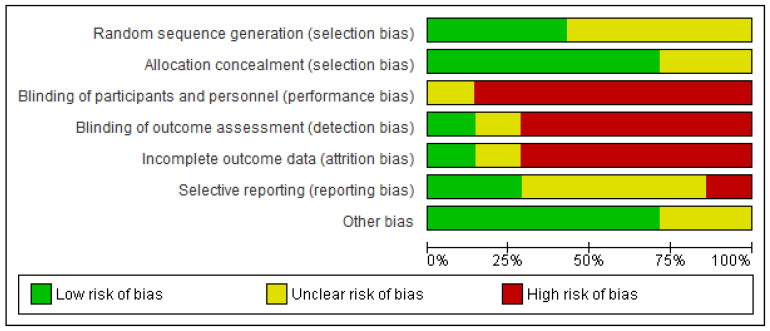
Risk of bias graph.

**Figure 3:**
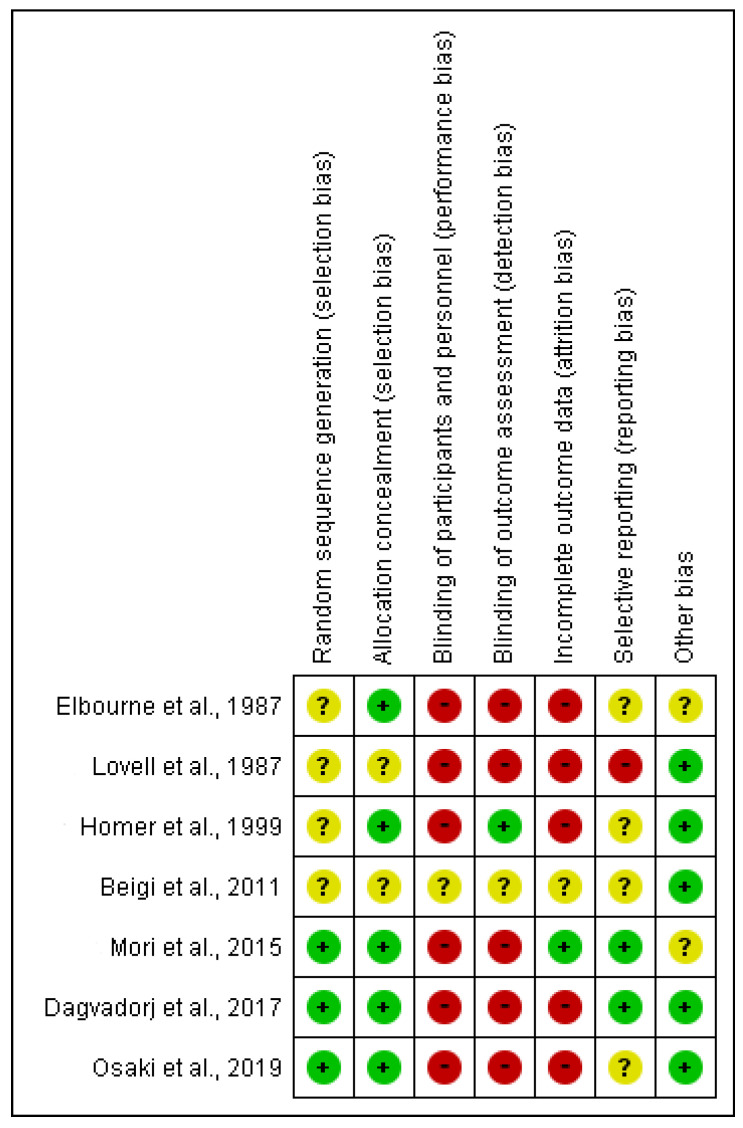
Risk of bias summary [19,20,21,22,23,24,25]. Red color with a − symbol means a high risk of bias, yellow color with a ? symbol means an unclear risk of bias, and green color with a + symbol means a low risk of bias.

All studies showed some concerns in most of the domains of the Cochrane risk-of-bias assessment tool (Figure 2). Four RCTs had an unclear risk of bias for random sequence generation (Figure 3). The performance bias and detection bias were high in most RCTs. Only one RCT had a low risk of bias for incomplete outcome data, with the unclear remainder (one RCT) or high risk of bias (five RCTs). The risk of reporting bias was high or unclear in more than half of the RCTs. The other risk of bias was low in most RCTs.

### 3.4. Pooled Effects of MCH Handbook Interventions on Improving Maternal, Newborn, and Child Health Outcomes

#### 3.4.1. Number of ANC Visits

**Figure 4:**
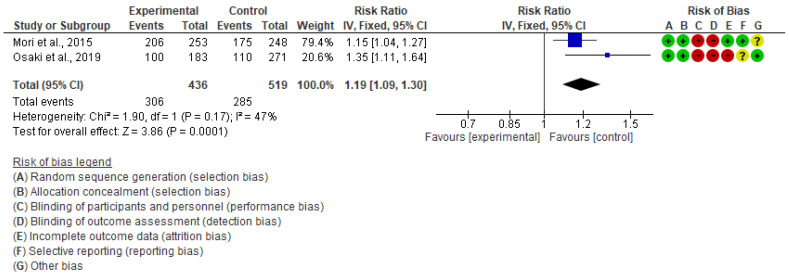
Meta-analysis for the effect of MCH handbook interventions vs. standard care on ≥6 ANC visits [19,20]. Red color with a − symbol means a high risk of bias, yellow color with a ? symbol means an unclear risk of bias, and green color with a + symbol means a low risk of bias.

**Figure 5:**
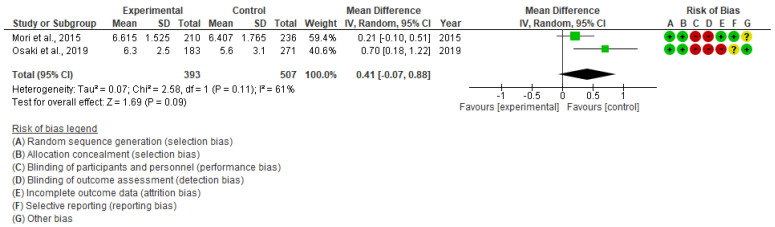
Meta-analysis for the effect of MCH handbook interventions vs. standard care on mean number of ANC visits [19,20]. Red color with a − symbol means a high risk of bias, yellow color with a ? symbol means an unclear risk of bias, and green color with a + symbol means a low risk of bias.

We performed an inverse variance random-effects meta-analysis to summarize the effect on uptake of ANC visit utilization among pregnant women. A total of 2 studies, consisting of 1148 pregnant women, have reported this outcome [19,20]. The pooled estimates showed that the risk of ≥6 ANC visit utilization among pregnant women was 19% higher in the intervention group than in the control group (RR 1.19, 95% CI: 1.09 to 1.30, I^2^ = 47%, 2 studies, 955 women, moderate certainty of evidence) (Figure 4). However, there may be negligible differences between intervention and control groups regarding the mean number of ANC visits (MD 0.41, 95% CI: −0.07 to 0.88, I^2^ = 61%, 2 studies, 900 women, very low certainty of evidence) (Figure 5). With respect to immunization during pregnancy, one study reported tetanus toxoid injection uptake outcome and found a positive effect on the uptake of the injection than in the control group (OR 1.98, 95% CI: 1.29 to 3.04) [20].

#### 3.4.2. Skilled Birth Attendance at the Time of Delivery

One study reported the outcome of skilled birth attendance during delivery [20], and there was no difference between the intervention and control groups (RR 1.10, 95% CI: 0.88 to 1.38). For continuum of care from prenatal to postnatal, an Indonesian study found the risk of the continuum of care uptake was 138% higher in the intervention group than in the control group (OR 2.38, 95% CI: 1.22 to 4.64) [20].

#### 3.4.3. Cesarean Delivery

**Figure 6:**
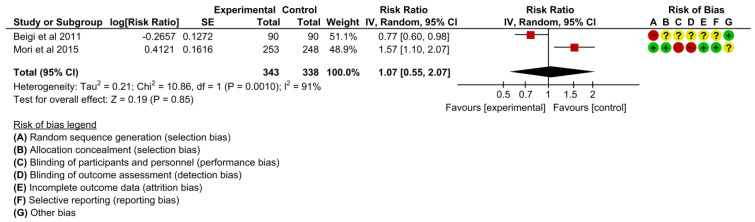
Meta-analysis for the effect of MCH handbook interventions vs. standard care on cesarean delivery [19,21]. Red color with a − symbol means a high risk of bias, yellow color with a ? symbol means an unclear risk of bias, and green color with a + symbol means a low risk of bias.

Two studies reported cesarean delivery [19,21]. The effects on cesarean delivery were unclear during pooled effect under meta-analysis (RR 1.07, 95% CI: 0.55 to 2.07, I^2^ = 91%, 2 studies, 681 women, very low certainty of evidence) (Figure 6).

#### 3.4.4. Breastfeeding Practice

**Figure 7:**
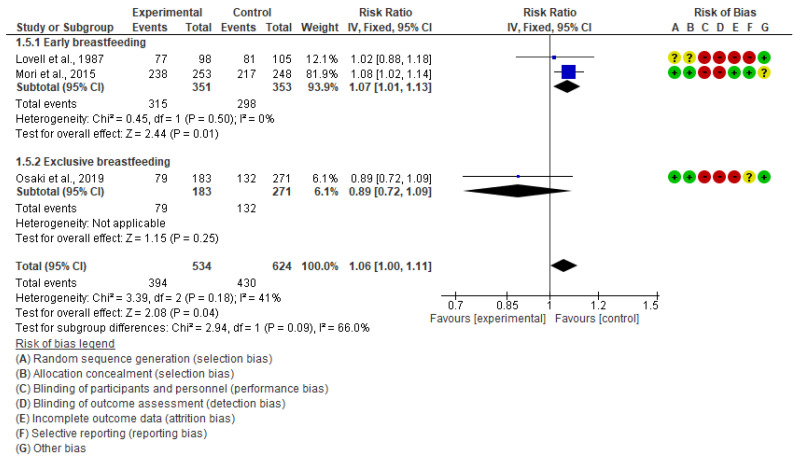
Meta-analysis for the effect of MCH handbook interventions vs. standard care on breastfeeding practice [19,20,22]. Red color with a − symbol means a high risk of bias, yellow color with a ? symbol means an unclear risk of bias, and green color with a + symbol means a low risk of bias.

Three studies assessed the effectiveness on breastfeeding practice outcomes [19,20,22]. We conducted a subgroup analysis considering early breastfeeding and exclusive breastfeeding practices and found a positive effect of interventions on early breastfeeding practice (RR 1.07, 95% CI: 1.01 to 1.13, I^2^ = 0%, 2 studies, 704 women, moderate certainty of evidence) (Figure 7). However, there may be little difference between the intervention and control groups regarding improving exclusive breastfeeding practice (RR 0.89, 95% CI: 0.72 to 1.09, 1 study, 454 women, very low certainty of evidence) or overall breastfeeding practice (RR 1.03, 95% CI: 0.94 to 1.13, I^2^ = 41%, 3 studies, 1158 women, moderate certainty of evidence).

#### 3.4.5. Maternal Smoking

**Figure 8:**
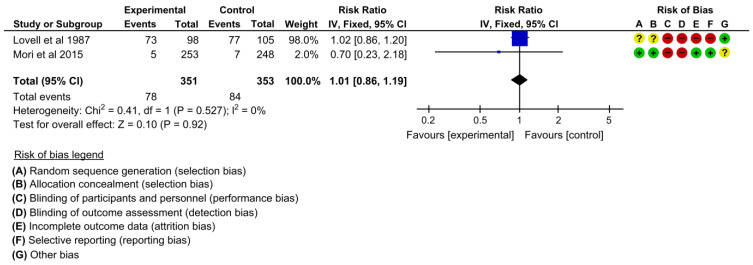
Meta-analysis for the effect of MCH handbook interventions vs. standard care on maternal smoking [19,22]. Red color with a − symbol means a high risk of bias, yellow color with a ? symbol means an unclear risk of bias, and green color with a + symbol means a low risk of bias.

Two studies assessed the effectiveness on reducing maternal smoking [19,22]. There was little difference between the intervention and control groups regarding reducing maternal smoking when we pooled the effects in the meta-analysis (RR 1.01, 95% CI: 0.86 to 1.19, I^2^ = 0%, 2 studies, 704 women, moderate certainty of evidence) (Figure 8).

Smoking outcome among other members of the household during pregnancy was reported in one study [19]. The intervention slightly reduced smoking among other members of the household during pregnancy (RR 0.84, 95% CI: 0.71 to 0.99).

#### 3.4.6. Infant Morbidity and Mortality

**Figure 9:**
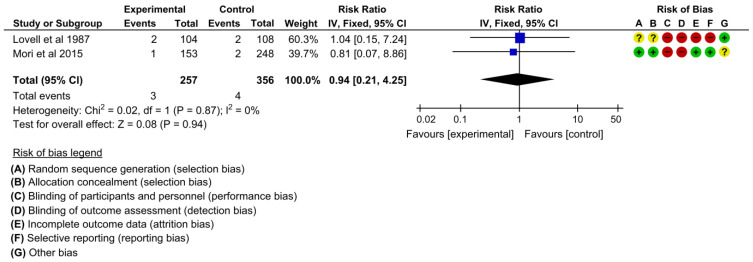
Meta-analysis for the effect of MCH handbook interventions vs. standard care on stillbirth or neonatal death [19,22]. Red color with a − symbol means a high risk of bias, yellow color with a ? symbol means an unclear risk of bias, and green color with a + symbol means a low risk of bias.

Two studies assessed the effectiveness on reducing stillbirth or neonatal deaths [19,22]. The pooled estimates showed no difference between the intervention and control groups regarding reducing stillbirth or neonatal deaths (RR 0.94, 95% CI: 0.21 to 4.25, I^2^ = 0%, 2 studies, 613 women, low certainty of evidence) (Figure 9).

One study reported preterm labor, and the intervention group had a lower frequency of preterm labor (*p* = 0.015) [21]. In the Mongolian study [19], there was no difference between the intervention and control groups for outcomes of congenital malformation (RR 1.96, 95% CI: 0.49 to 7.75); newborns’ birth weight (MD −40.50, 95% CI: −141.53 to 60.53, *p* = 0.432); Apgar score in 5 min (MD 0.21, 95% CI: −0.21 to 0.63); and neonatal admission to NICU (RR 1.18, 95% CI: 0.36 to 3.8).

One study in Indonesia reported vitamin A uptake outcome and found a positive effect on the uptake of vitamin A than in the control group (OR 2.0, 95% CI: 1.16 to 3.47) [20].

The Indonesian study reported stunting, wasting, and underweight observed in children [20]. The study found a positive effect on reducing the proportion of underweight (OR 0.33, 95% CI: 0.12 to 0.94) and stunted children (OR 0.53, 95% CI: 0.30 to 0.92), but there may be little difference between the intervention and control groups in terms of reducing wasting of children (OR 0.59, 95% CI: 0.24 to 1.47). Another study in Mongolia reported cognitive developmental delay and found that the risk of developing cognitive developmental delay was 68% lower in the intervention group than in the control group (OR 0.32, 95% CI: 0.14 to 0.73, *p* = 0.007) [23].

#### 3.4.7. Drinking during Pregnancy

**Figure 10:**
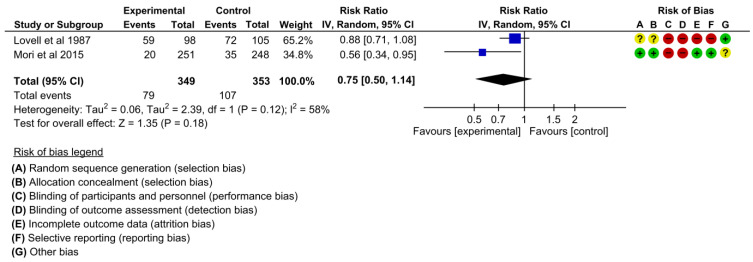
Meta-analysis for the effect of MCH handbook interventions vs. standard care on drinking during pregnancy [19,22]. Red color with a − symbol means a high risk of bias, yellow color with a ? symbol means an unclear risk of bias, and green color with a + symbol means a low risk of bias.

Two studies assessed the effectiveness on maternal drinking behavior during pregnancy [19,22]. There may be little difference between the intervention and control groups regarding reducing drinking behavior during pregnancy when we pooled the effects in the meta-analysis (RR 0.75, 95% CI: 0.50 to 1.14, I^2^ = 58%, 2 studies, 702 women, low certainty of evidence) (Figure 10).

#### 3.4.8. Case Notes Lost or Left at Home

**Figure 11:**
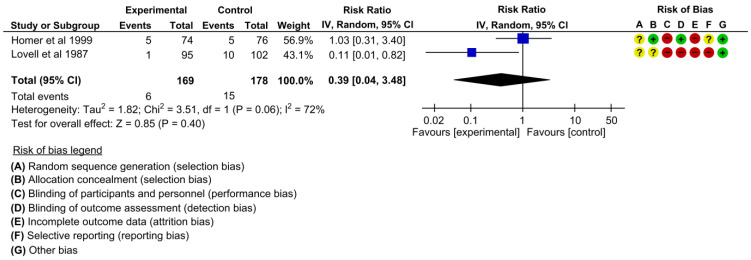
Meta-analysis for the effect of MCH handbook interventions vs. standard care on case notes lost or left at home [22,24]. Red color with a − symbol means a high risk of bias, yellow color with a ? symbol means an unclear risk of bias, and green color with a + symbol means a low risk of bias.

Two studies assessed the effectiveness on outcomes of case notes lost or left at home [22,24]. The pooled estimates showed little difference between the intervention and control groups regarding case notes lost or left at home when we pooled the effects in the meta-analysis (RR 0.39, 95% CI: 0.04 to 3.48, I^2^ = 72%, 2 studies, 347 women, very low certainty of evidence) (Figure 11).

#### 3.4.9. Women Satisfied with Antenatal or Maternity Care

**Figure 12:**
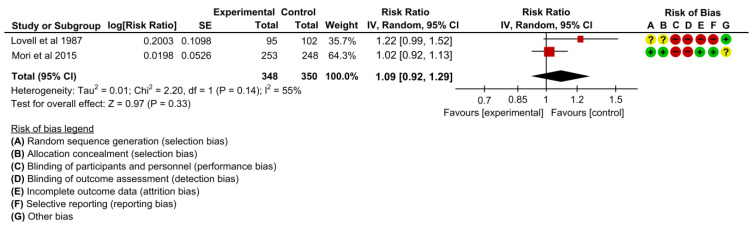
Meta-analysis for the effect of MCH handbook interventions vs. standard care on women satisfied with antenatal or maternity care [19,22]. Red color with a − symbol means a high risk of bias, yellow color with a ? symbol means an unclear risk of bias, and green color with a + symbol means a low risk of bias.

Two studies assessed the effectiveness on women satisfied with antenatal or maternity care [19,22]. There may be little difference between the intervention and control groups regarding improving women’s satisfaction with antenatal or maternity care when we pooled the effects in the meta-analysis (RR 1.09, 95% CI: 0.92 to 1.29, I2 = 55%, 2 studies, 698 women, low certainty of evidence) (Figure 12).

#### 3.4.10. Women Who Wanted to Carry Their Case Notes in Subsequent Pregnancy

**Figure 13:**
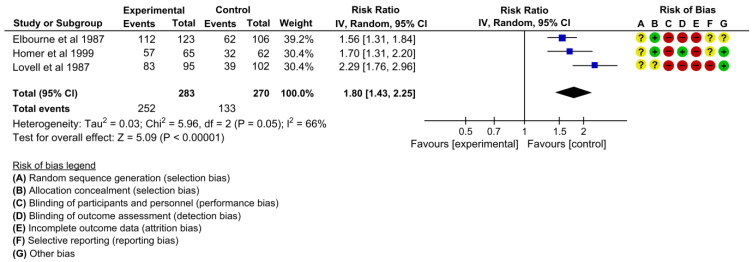
Meta-analysis for the effect of MCH handbook interventions vs. standard care on women who wanted to carry their case notes in subsequent pregnancy [22,24,25]. Red color with a − symbol means a high risk of bias, yellow color with a ? symbol means an unclear risk of bias, and green color with a + symbol means a low risk of bias.

Three studies assessed the effectiveness on the outcome of women who wanted to carry their case notes in the subsequent pregnancy [22,24,25]. The pooled estimates showed that the proportion of women who wanted to carry their case notes in the subsequent pregnancy was 80% higher in the intervention group than in the control group (RR 1.80, 95% CI: 1.43 to 2.25, I^2^ = 66%, 3 studies, 553 women, low certainty of evidence) (Figure 13).

#### 3.4.11. Women Who Felt in Control during ANC

**Figure 14:**
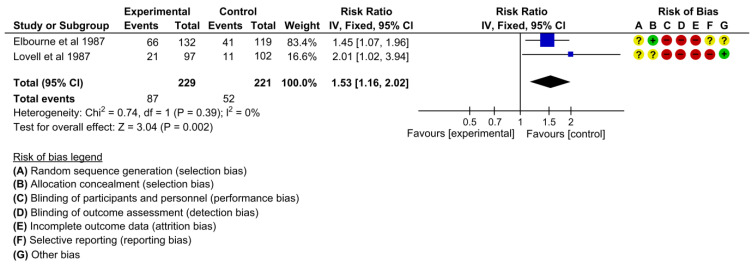
Meta-analysis for the effect of MCH handbook interventions vs. standard care on women felt in control during ANC [22,25]. Red color with a − symbol means a high risk of bias, yellow color with a ? symbol means an unclear risk of bias, and green color with a + symbol means a low risk of bias.

Two studies assessed the effectiveness on the outcome of women who felt in control during ANC [22,25]. The pooled estimates showed that the proportion of women who felt in control during ANC was 53% higher in the intervention group than in the control group (RR 1.53, 95% CI: 1.16 to 2.02, I^2^ = 0%, 2 studies, 450 women, moderate certainty of evidence) (Figure 14). One study reported women who felt they could talk more easily with doctors and midwives [25]. The proportion of women who felt they could talk more easily with doctors and midwives was higher in the intervention group than in the control group (RR 1.73, 95% CI: 1.16 to 2.59).

#### 3.4.12. Support from Family during Pregnancy

**Figure 15:**
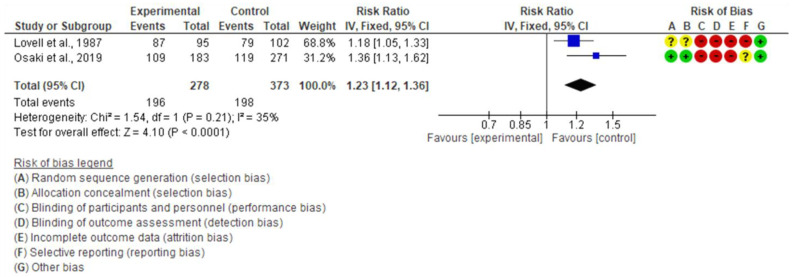
Meta-analysis for the effect of MCH handbook interventions vs. standard care on support from family during pregnancy [20,22]. Red color with a − symbol means a high risk of bias, yellow color with a ? symbol means an unclear risk of bias, and green color with a + symbol means a low risk of bias.

Two studies assessed the effectiveness on the outcome of women who had family support during their pregnancy [20,22]. As for the type of support, the trial conducted in Indonesia provided financial support during pregnancy, whereas that in the UK provided companionship during labor. The pooled estimates showed that the proportion of women who had their husbands’ support during their pregnancy was 25% higher in the intervention group than in the control group (RR 1.23, 95% CI: 1.12 to 1.36, I^2^ = 35%, 2 studies, 651 women, low certainty of evidence) (Figure 15).

#### 3.4.13. Maternal Morbidity and Mortality

One study reported preeclampsia and found little difference between intervention and control groups [21]. One study in Mongolia found a positive effect identifying complications during pregnancy (RR 2.17, 95% CI: 1.18 to 3.98), but no difference between intervention and control groups for maternal admission to ICU during pregnancy (RR 0.32, 95% CI: 0.03 to 3.1) [19].

As for postpartum maternal health, one study reported postpartum infection and found a correlation between intervention and control groups (*p* = 0.012) [21]. A Mongolian study reported postnatal depression using the 12 cutoff points of the Edinburgh Postnatal Depression Scale (EPDS) [19,26], and no difference was found between the intervention and control groups (RR 0.99, 95% CI: 0.94 to 1.04). However, another study in Iran evaluated the effectiveness on quality of life after 42 days of delivery and found a better quality of life in the intervention group than the control group (*p* = 0.026 in physical and mental dimensions; *p* = 0.049 in physical dimensions; *p* = 0.02 in mental dimensions) [21].

The Indonesian study reported care-seeking for pregnancy and postpartum complications and newborn illnesses [20]. There may be little difference between the intervention and control groups regarding care-seeking for pregnancy complications (OR 2.6, 95% CI: 0.52 to 13.04), postpartum complications (OR 5.0, 95% CI: 0.76 to 32.93), and newborn illnesses (OR 1.76, 95% CI: 0.45 to 6.98).

#### 3.4.14. Certainty of the Evidence

Table 1 shows the certainty of evidence for the effectiveness of the MCH handbook on outcomes.

## 4. Discussion

This systematic review revealed the MCH handbook improves maternal health service utilization, such as more frequent ANC visits, and earlier initiation of breastfeeding compared to the control group. In studies conducted in Mongolia and Indonesia, more women in the intervention group with the MCH handbook attended more than six ANC visits compared with the control group. The MCH handbook includes advice on MCH, which could lead to improved knowledge of pregnancy and childbirth, awareness of MCH problems, and the use of healthcare services [3,19]. Regarding the number of ANC visits, the WHO guidelines published in 2016 recommend a minimum of eight contacts to prevent perinatal deaths, and the effectiveness of the MCH handbook for more than eight contacts should also be investigated [27,28]. In addition to the Indonesian study, there was also a controlled trial conducted in Cambodia on childbirth by skilled health personnel [29]. However, the Cambodian study was considered a quasi-randomized controlled trial and was not included in the present review.

Although the Indonesian study found no difference between intervention and control groups regarding exclusive breastfeeding for the first 6 months after delivery [20], other studies conducted in the United Kingdom and Mongolia found that more women in the intervention group performed early initiation of breastfeeding compared with the control group [19,22]. A systematic review published in 2015 reported that late initiation of breastfeeding after the first hour of life is associated with an increased risk of neonatal death [30]. Therefore, early initiation of breastfeeding improves the newborn’s health status, and the distribution of MCH handbooks may contribute to the well-being of newborns.

In the previous systematic review, the results of one RCT showed an increase in cesarean sections in the intervention group [9]. In this systematic review, a meta-analysis of two trials showed no difference in cesarean sections between the intervention and control groups. Although the trial conducted in Iran showed women in the intervention group are less likely to receive cesarean sections [21], another trial in Mongolia showed that providing an MCH handbook probably increases this outcome slightly [19]. To our knowledge, this is the first time a trial conducted in Iran has been included in systematic reviews of home-based records for MCH.

We have observed positive effects of carrying health records on the sense of control during ANC and communication with healthcare providers [22,25]. The home-based records benefit women by allowing easy access to their data on MCH at home [4]. These records help pregnant women track their health status and increase their autonomy during the pregnancy period [9]. Additionally, carrying their record not only promotes communication between healthcare providers and pregnant women but also increases the sense of sharing and communication among family members [24,31].

One of the limitations of this systematic review is the small number of included studies. No more than two trials were available for some outcomes, hindering the ability to perform meta-analysis. Another limitation was the high risk of bias for the included studies, especially performance bias. In addition, most included studies did not provide clear information on whether random sequence generation was performed. Finally, there was another limitation regarding the inclusion of all forms of MCH handbooks as interventions, entailing that the included studies differed in their intervention-related methodologies.

## 5. Conclusions

The MCH handbook can increase the utilization of maternal health services, such as ANC visits, and early initiation of breastfeeding compared with the control group. It also leads to a sense of autonomy during ANC, better communication with healthcare providers, and support from family members. However, some of the included studies in this review have high risk of bias or unclear risk of bias, which requires caution in interpreting the results. Additionally, with technological developments, some countries have introduced electronic MCH handbooks, and thus, studies on the electronic MCH handbook will need to be evaluated in the future.

## Figures and Tables

**Table 1:** Summary of findings.

Outcomes	Anticipated Absolute Effects * (95% CI)		Relative Effect (95% CI)	No. of Participants (Studies)	Certainty of the Evidence (GRADE)	Comments
	Risk with Standard Care or No MCH Handbook	Risk with MCH Handbook				
≥6 ANC visits	549 per 1000	653 per 1000 (599 to 714)	RR 1.19 (1.09 to 1.30)	955 (2 RCTs)	⨁⨁⨁◯ MODERATE	Inverse variance
Cesarean delivery	0 per 1000	0 per 1000 (0 to 0)	RR 1.07 (0.55 to 2.07)	681 (2 RCTs)	⨁◯◯◯ VERY LOW	Inverse variance
Early breastfeeding	844 per 1000	903 per 1000 (853 to 954)	RR 1.07 (1.01 to 1.13)	704 (2 RCTs)	⨁⨁⨁◯ MODERATE	Inverse variance
Women who wanted to carry their case notes in subsequent pregnancy	493 per 1000	887 per 1000 (704 to 1000)	RR 1.80 (1.43 to 2.25)	553 (3 RCTs)	⨁⨁◯◯ LOW	Inverse variance
Women felt in control during ANC	235 per 1000	360 per 1000 (273 to 475)	RR 1.53 (1.16 to 2.02)	450 (2 RCTs)	⨁⨁⨁◯ MODERATE	Inverse variance
Family support during pregnancy	531 per 1000	653 per 1000 (595 to 722)	RR 1.23 (1.12 to 1.36)	651 (2 RCTs)	⨁⨁◯◯ LOW	Inverse variance

Notes: Patient or population: pregnant women or mother. Setting: Australia, Cambodia, Indonesia, Iran, Mongolia, and the UK. Intervention: MCH handbook. Comparison: standard care or no MCH handbook. * Risk in the intervention group (and its 95% confidence interval) is based on the assumed risk in the comparison group and the relative effect of the intervention (and its 95% CI). CI, confidence interval; RR, risk ratio; GRADE, Grading of Recommendations Assessment, Development, and Evaluation; MCH, maternal and child health; ANC, antenatal care; RCT, randomized control trials; SBA, skilled birth attendance. GRADE Working Group grades of evidence: high certainty = we are very confident that the true effect lies close to that of the estimate of the effect; moderate certainty = we are moderately confident in the effect estimate: the true effect is likely to be close to the estimate of the effect, but there is a possibility that it is substantially different; low certainty = our confidence in the effect estimate is limited: the true effect may be substantially different from the estimate of the effect; very low certainty = we have very little confidence in the effect estimate: the true effect is likely to be substantially different from the estimate of the effect.

## Data Availability

All data were obtained from published journal articles. The extracted data are available upon reasonable request to the corresponding author.

## Supplementary Materials

The following supporting information can be downloaded at: https://www.mdpi.com/article/10.3390/children10030435/s1, Table S1: PRISMA 2020 Checklist; Table S2: Search strategy; Table S3: Characteristics of the included studies.
