# Supplementary material for: Role of Maternal and Child Health Handbook on Improving Maternal, Newborn, and Child Health Outcomes: A Systematic Review and Meta-Analysis
Source: Children (Basel). 2023 Feb 23;10(3):435. doi: 10.3390/children10030435 (PMC10046926; doi:10.3390/children10030435)
Supplement: Supplementary file 1 [file children-10-00435-s001.zip › children-2180030-supplementary.pdf]

**Supplementary Materials:**

**Table S1.** PRISMA 2020 Checklist.

| Section and Topic | Item # | Checklist item | Location where item is reported |
|---|---|---|---|
| **TITLE** | | | |
| Title | 1 | Identify the report as a systematic review. | 1 |
| **ABSTRACT** | | | |
| Abstract | 2 | See the PRISMA 2020 for Abstracts checklist. | 1 |
| **INTRODUCTION** | | | |
| Rationale | 3 | Describe the rationale for the review in the context of existing knowledge. | 1-2 |
| Objectives | 4 | Provide an explicit statement of the objective(s) or question(s) the review addresses. | 2 |
| **METHODS** | | | |
| Eligibility criteria | 5 | Specify the inclusion and exclusion criteria for the review and how studies were grouped for the syntheses. | 2 |
| Information sources | 6 | Specify all databases, registers, websites, organisations, reference lists and other sources searched or consulted to identify studies. Specify the date when each source was last searched or consulted. | 3 |
| Search strategy | 7 | Present the full search strategies for all databases, registers and websites, including any filters and limits used. | supplementary materials, 3-4 |
| Selection process | 8 | Specify the methods used to decide whether a study met the inclusion criteria of the review, including how many reviewers screened each record and each report retrieved, whether they worked independently, and if applicable, details of automation tools used in the process. | 3-4 |
| Data collection process | 9 | Specify the methods used to collect data from reports, including how many reviewers collected data from each report, whether they worked independently, any processes for obtaining or confirming data from study investigators, and if applicable, details of automation tools used in the process. | 3-4 |
| Data items | 10a | List and define all outcomes for which data were sought. Specify whether all results that were compatible with each outcome domain in each study were sought (e.g. for all measures, time points, analyses), and if not, the methods used to decide which results to collect. | 3 |

| Section and Topic | Item # | Checklist item | Location where item is reported |
|---|---|---|---|
| | 10b | List and define all other variables for which data were sought (e.g. participant and intervention characteristics, funding sources). Describe any assumptions made about any missing or unclear information. | 2 |
| Study risk of bias assessment | 11 | Specify the methods used to assess risk of bias in the included studies, including details of the tool(s) used, how many reviewers assessed each study and whether they worked independently, and if applicable, details of automation tools used in the process. | 4 |
| Effect measures | 12 | Specify for each outcome the effect measure(s) (e.g. risk ratio, mean difference) used in the synthesis or presentation of results. | 4 |
| Synthesis methods | 13a | Describe the processes used to decide which studies were eligible for each synthesis (e.g. tabulating the study intervention characteristics and comparing against the planned groups for each synthesis (item #5)). | 4 |
| | 13b | Describe any methods required to prepare the data for presentation or synthesis, such as handling of missing summary statistics, or data conversions. | 4 |
| | 13c | Describe any methods used to tabulate or visually display results of individual studies and syntheses. | 4 |
| | 13d | Describe any methods used to synthesize results and provide a rationale for the choice(s). If meta-analysis was performed, describe the model(s), method(s) to identify the presence and extent of statistical heterogeneity, and software package(s) used. | 4 |
| | 13e | Describe any methods used to explore possible causes of heterogeneity among study results (e.g. subgroup analysis, meta-regression). | 4 |
| | 13f | Describe any sensitivity analyses conducted to assess robustness of the synthesized results. | 4 |
| Reporting bias assessment | 14 | Describe any methods used to assess risk of bias due to missing results in a synthesis (arising from reporting biases). | 4 |
| Certainty assessment | 15 | Describe any methods used to assess certainty (or confidence) in the body of evidence for an outcome. | 4 |
| **RESULTS** | | | |
| Study selection | 16a | Describe the results of the search and selection process, from the number of records identified in the search to the number of studies included in the review, ideally using a flow diagram. | 5, Figure 1 |
| | 16b | Cite studies that might appear to meet the inclusion criteria, but which were excluded, and explain why they were excluded. | 5 |

| Section and Topic | Item # | Checklist item | Location where item is reported |
|---|---|---|---|
| Study characteristics | 17 | Cite each included study and present its characteristics. | supplementary materials, 5 |
| Risk of bias in studies | 18 | Present assessments of risk of bias for each included study. | 5-6, Figure 3 |
| Results of individual studies | 19 | For all outcomes, present, for each study: (a) summary statistics for each group (where appropriate) and (b) an effect estimate and its precision (e.g. confidence/credible interval), ideally using structured tables or plots. | 7-14 |
| Results of syntheses | 20a | For each synthesis, briefly summarise the characteristics and risk of bias among contributing studies. | 7-14 |
| | 20b | Present results of all statistical syntheses conducted. If meta-analysis was done, present for each the summary estimate and its precision (e.g. confidence/credible interval) and measures of statistical heterogeneity. If comparing groups, describe the direction of the effect. | 7-14 |
| | 20c | Present results of all investigations of possible causes of heterogeneity among study results. | Not applicable |
| | 20d | Present results of all sensitivity analyses conducted to assess the robustness of the synthesized results. | Not applicable |
| Reporting biases | 21 | Present assessments of risk of bias due to missing results (arising from reporting biases) for each synthesis assessed. | 5-6 |
| Certainty of evidence | 22 | Present assessments of certainty (or confidence) in the body of evidence for each outcome assessed. | 14 |
| **DISCUSSION** | | | |
| Discussion | 23a | Provide a general interpretation of the results in the context of other evidence. | 15 |
| | 23b | Discuss any limitations of the evidence included in the review. | 15 |
| | 23c | Discuss any limitations of the review processes used. | 15 |
| | 23d | Discuss implications of the results for practice, policy, and future research. | 15 |
| **OTHER INFORMATION** | | | |

| Section and Topic | Item # | Checklist item | Location where item is reported |
|---|---|---|---|
| Registration and protocol | 24a | Provide registration information for the review, including register name and registration number, or state that the review was not registered. | 2 |
| | 24b | Indicate where the review protocol can be accessed, or state that a protocol was not prepared. | 2 |
| | 24c | Describe and explain any amendments to information provided at registration or in the protocol. | Not applicable |
| Support | 25 | Describe sources of financial or non-financial support for the review, and the role of the funders or sponsors in the review. | 16 |
| Competing interests | 26 | Declare any competing interests of review authors. | 16 |
| Availability of data, code and other materials | 27 | Report which of the following are publicly available and where they can be found: template data collection forms; data extracted from included studies; data used for all analyses; analytic code; any other materials used in the review. | 16 |

*From:* Page MJ, McKenzie JE, Bossuyt PM, Boutron I, Hoffmann TC, Mulrow CD, et al. The PRISMA 2020 statement: an updated guideline for reporting systematic reviews. BMJ 2021;372:n71. doi: 10.1136/bmj.n71

For more information, visit: http://www.prisma-statement.org/

**Table S2**. Search strategy.

| SL | Search terms |
|---|---|
| #1 | Population-<br>• expectant mother OR mother OR pregnant women OR pregnant mother OR pregnancy<br>• child OR children OR newborn OR neonate OR infant |
| #2 | Intervention-<br>• maternal and child health handbook OR MCH handbook OR maternal and child health book OR MCH book OR maternal and child health booklet OR MCH booklet OR maternal and child health card OR MCH card OR maternal and child health record OR MCH record OR record book<br>• paper-based record OR home-based record OR electronic record OR personal health record<br>• child vaccination card OR child immunization card OR child health record OR child health book OR child health booklet OR child health handbook<br>• antenatal handbook OR antenatal book OR antenatal booklet OR antenatal card OR antenatal record<br>• prenatal handbook OR prenatal book OR prenatal booklet OR prenatal card OR prenatal record<br>• postnatal handbook OR postnatal book OR postnatal booklet OR postnatal card OR postnatal record |
| #3 | Study design-<br>• randomized controlled trials OR quasi-randomized controlled trials OR cluster-randomized controlled trials |
| #4 | #1 AND #2 AND #3 |

**Table S3.** Characteristics of the included studies.

| Author, Year | Country | Study design | Participants | Sample size | Intervention | Comparator |
|---|---|---|---|---|---|---|
| Elbourne et al., 1987 | United Kingdom | Individual randomized controlled trial | Women less than 34 weeks gestation who booked for perinatal care with E.H. at the peripheral clinic at the Sandleford Hospital, Xewbury from 1 January to 30 June 1984. | 290 | To hold their own obstetric case notes until 10 days after delivery. | To hold a co-operation card while their case notes were held by the medical records department. |
| Lovell et al., 1987 | United Kingdom | Individual randomized controlled trial | Women 21 weeks gestation from 20 June to 7 November 1984.<br>Age mean (SD): 26.4 years (6.1) in intervention group, 26.9 years (5.7) in control group. | 235 | To hold their maternity case notes until admission to hospital for delivery | To hold their co-operation cards and their maternity case notes were retained by the hospital in the usual way. |
| Homer et al., 1999 | Australia | Individual randomized controlled trial | English-speaking women visiting the hospital clinic for their first antenatal care.<br>Age at entry: 28.6 years (SD 5.3) in intervention group, 28.8 years (SD 5.4) in control group.<br>Gestation at enrolment: 16.6 weeks (SD 4.6) in intervention group, 16.7 weeks (SD 4.2) in control group. | 128 | To hold the hospital antenatal record, and the cooperation card was retained at the hospital as a replacement in case the woman lost her records. | To hold their cooperation card.<br>The complete set of antenatal records was retained by the hospital and retrieved at each antenatal visit. |

| | | | | | | |
|---|---|---|---|---|---|---|
| Beigi et al., 2011 | Iran | Individual randomized controlled trial | Women with at least three pregnancies and women whose referral was before the second trimester of pregnancy.<br>Mean age of the mothers in intervention and control groups was 26.5 and 27.3 years, respectively. | 180 | To be provided comprehensive maternal care log book designed by the researchers (including pages of records and education) | To hold pregnancy card provided by physicians or midwives (simple maternal cards) |
| Mori et al., 2015 | Mongolia | Cluster randomized controlled trial | Pregnant women living in the Bulgan province of Mongolia.<br>Mean maternal age (SD): 27.3 years (6.13) in intervention group, 27.7 years (5.67) in control group.<br>A cluster-randomized controlled study was conducted from 1 May 2009 until 1 September<br>2010 among pregnant women and their infants who lived in Bulgan, Mongolia.<br>Eligible participants included | 501 | To be provided the handbook translated into Mongolian from the original Japanese version.<br><br>(The handbooks were implemented at the beginning of the study observational period.) | Without intervention (The intervention was delayed by seven months.) |
| Dagvadorj et al., 2017 (Three-year follow-up study of Mori et al., 2015) | Mongolia | Cluster randomized controlled trial | All women living in the Bulgan province of Mongolia who gave birth between March and August 2010 participated in the study. If they still lived in the area between July and October 2013, they were the participants of the three-year follow-up study. | 386 | | |

| | | | | | | |
|---|---|---|---|---|---|---|
| Osaki et al., 2019 | Indonesia | Cluster randomized controlled trial | Women attending a selected health center in Garut district.<br>Average age (years): 27.2 years (7.7) in intervention group, 26.8 years (6.4) in control group.<br>(Data were collected from January through March 2007 and 2009 (with a 2-year follow-up)). | 647 | Intervention included<br>(i) to be provided maternal and child health handbooks at antenatal checkups,<br>(ii) to be provided with records in the handbooks and instructions on using the handbooks by health staff, and<br>(iii) to receive an explanation from volunteers about the use of healthcare services and home care using the handbooks. | Without intervention |
